# Optimization-Based Economical Flexural Design of Singly Reinforced Concrete Beams: A Parametric Study

**DOI:** 10.3390/ma15093223

**Published:** 2022-04-29

**Authors:** Rizwan Azam, Muhammad Rizwan Riaz, Muhammad Umer Farooq, Faraz Ali, Muhammad Mohsan, Ahmed Farouk Deifalla, Abdeliazim Mustafa Mohamed

**Affiliations:** 1 Department of Civil Engineering, University of Engineering and Technology, Lahore 54890, Pakistan; azam.rizwan@uet.edu.pk (R.A.); domain479@gmail.com (F.A.); mr.mughal75@gmail.com (M.M.); 2 Department of Civil Engineering, Khwaja Fareed University of Engineering and Information Technology, Rahim Yar Khan 64200, Pakistan; mumer.farooq@kfueit.edu.pk; 3 Structural Engineering and Construction Management Department, Future University in Egypt, Cairo 11835, Egypt; ahmed.deifalla@fue.edu.eg; 4 Department of Civil Engineering, College of Engineering, Prince Sattam bin Abdulaziz University, Al-Kharj 12673, Saudi Arabia; a.bilal@psau.edu.sa

**Keywords:** optimization tool, reinforced concrete beam, optimization techniques, spreadsheet optimization, Evolutionary Algorithm, optimum design

## Abstract

In the past, many studies have been conducted on the optimization of reinforced concrete (RC) structures. These studies have demonstrated the effectiveness of different optimization techniques to obtain an economical design. However, the use of optimization techniques to an obtain economical design is not so practical due to the difficulty in applying most of the optimization techniques to achieve an optimal solution. The RC beam is one of the most common structural elements encountered by a practising design engineer. The current study is designed to highlight the potential of the Solver tool in MS Excel as an easy-to-use option for optimizing the design of simply supported RC beams. A user-friendly interface was developed in a spreadsheet in which beam design parameters from a typical design can be entered and an economical design can be obtained using the Evolutionary Algorithm available in the MS Excel Solver tool. To demonstrate the effectiveness of the developed optimization tool, three examples obtained from the literature have been optimized. The results showed that up to 24% economical solution can be obtained by keeping the same material strengths that were assumed in the original design. However, if material strength is also considered as a variable, up to 44% of the economical solution can be obtained. A parametric study was also conducted to investigate the effect of different design variables on the economical design of simply supported RC beams and to derive useful rules of thumb for their design and proportioning, with the objective of cost minimization. The results of the parametric study suggest that the grade of the reinforcing steel is one of the most influential factors that affect the cost of simply supported RC beams. Practicing engineers can use the trends derived from this research to further refine their optimal designs.

## 1. Introduction

Optimization is the art of finding the best-suited candidate from a set of available options, without explicitly enumerating and evaluating all the possible alternatives [1]. The use of the optimization concept in concrete structures is for obtaining an economical cross-section of a reinforced concrete (RC) member that has sufficient capacity to counter the applied loading. The structural design engineers typically follow the iterative trial and error method to reach a feasible design option according to the applied loads. All this consumes a significant amount of time due to complicated calculations and does not always result in the most optimal and economical RC section [2]. For the design of RC members, the cost is not dependent on a single material, as is the case for steel construction; rather it, depends on the volumes of both concrete (cross-sectional dimensions) and reinforcing steel, making the optimization problem complex in nature. 

Different optimization methods have been used to solve different design problems in structural engineering. Some of these include the Generalized Reduced Gradient (GRG) [1] technique, Genetic Algorithms (GA) [3,4,5], Simulated Annealing (SA) [6], Sequential Quadratic Programming (SQP), Non-Linear Programming (NLP) [7], Harmony Search (HS) [1], and Particle Swarm Optimization (PSO) [8]. The optimization studies conducted using these methods have shown their effectiveness; however, their application in practical design is limited [3,9,10]. This is mainly because these techniques require the design engineer to possess in-depth knowledge of the optimization algorithms, the formulation of the optimization problem, and the coding of the design problems using programming languages. Hence, for the economical design of RC members, not only a proper understanding of optimization is necessary but also the right tool is a need of the hour due to the complex and lengthy nature of the problem.

The RC beam is one of the most common structural members that a design practitioner has to design, and it is encountered in the design of a wide range of structures, from a single-story masonry building to an RC skyscraper. Under ideal considerations, the whole structure should be considered in the optimization process, and its capital, operational, and maintenance costs must be taken into consideration. However, in most of the designs, this approach is not of practical use due to its complicated nature. Therefore, the optimization of individual structural members is generally preferred and adopted.

A significant amount of research has been published on the optimization of the design of an RC beam using different optimization approaches and algorithms. A state-of-the-art review on the optimal design of RC beams is presented by Rahmanian et al. [9] summarizing the design variables considered and the methods used by different researchers for the optimization of RC beams. The study also presents a spreadsheet implementation for the cost optimization of RC beams and performs a sensitivity analysis to investigate the effect of different design parameters such as the cost and strength of steel and concrete, the diameter of reinforcing bars, and the moment demand on the overall cost of RC beam. The study recommends an exhaustive enumeration method using the VBA code and finds that the higher strength of steel and the larger diameter of the steel bar greatly reduce the overall cost of an RC beam subjected to higher moment demand. Nigdeli et al. [11] used the Random Search Technique (RST) to find optimum cross-sectional dimensions and reinforcements of continuous RC beams. The internal forces of the RC beam were solved by using the three-moment equation for all iterations of RST. The design constraints given in ACI-318 [12] were satisfied, and the detailing of reinforcement bars was done in such a way as to promote maximum adherence. The presented approach was demonstrated with two numerical problems. The optimum results were obtained for different cross-section dimension ranges. Compared to the practice followed by designers, the presented approach was approximately 9% more economical for the two-span example. Thomas and Arulraj [13] worked on the optimization of RC beams using the Sequential Quadratic Programming (SQP) algorithm. They took steel and concrete strengths as design variables and found the efficiency of the SQP algorithm optimization to be good. Hisham Ajmal [14] studied the cost optimization of the singly reinforced RC beam designed as per ACI 318-08. The Genetic Algorithm (GA) optimization technique programmed in MATLAB was adopted. The different spans and imposed loads were considered, and the performance of the genetic algorithm was found to be satisfactory. Raouache et al. [15] worked on the optimization of three parameters of the RC beam, which are the strength of concrete, the spacing of stirrups, and the inclination angle of stirrups by using the Response Surface Methodology (RSM). They used the concept of the influence of parameters on the strength of the RC beam and found that the inclination of stirrups has more effect on the strength of the beam, followed by the spacing of stirrups and the strength of concrete. Correia et al. [16] investigated the effect of cross-sectional dimensions on the overall cost of the RC beam. They implemented the Evolutionary Algorithm (EA) in the Solver tool in MS Excel to optimize the beam design. Beams were designed according to the Brazilian standard [16]. They found the possibility of a 35% reduction in the optimized cost of the beam. Ozimboski et al. [17] optimized the cost of simply supported beams designed as per the Brazilian code using the Simulated Annealing (SA) optimization algorithm. They considered two different load levels and spans ranging from 1 to 25 m. It was observed that the difference in the optimal cost of RC beams regarding the minimum and maximum loads is lower than 15% and that the optimized dimensioning had the displacements limitation as to the active constraint for all spans and loads. Chutani and Singh [18] optimized the design of the RC beam using the Constriction Factor Particle Swarm Optimization (CFPSO) technique. Beam depth and percentage of reinforcement were considered as variables, and the optimization algorithm was coded in C++, which resulted in a 20% reduction in the cost of the beam.

The studies mentioned above have demonstrated the effectiveness of different optimization techniques to obtain an economical design for RC beams. However, the use of these optimization techniques is not so common in RC design because of the difficulty level associated with these techniques. There is a need for a simple tool that can be used to obtain optimal solutions without having in-depth knowledge of complex optimization algorithms, and the ability to code optimization problems in programming languages. In addition, there is a lack of a research study investigating the effect of the depth of the beam and the reinforcement ratio on the cost of the beam for different values of important parameters such as the commercially available grades of steel, concrete strength, and the required moment capacity for the beam. Hence, in the current study, a simple and user-friendly tool is developed in the familiar MS Excel, in which input parameters from a typical design as per ACI 318-19 [12] can be entered to obtain the most economical design. Further, as a novel objective, the developed tool is used to perform a parametric study to investigate the effect of the parameters such as the grades of steel and concrete, and the required moment capacity on the cost of the beam. The developed tool will be helpful in promoting design optimization among practising engineers, and the results of the parametric study will help them select such material grades that can further reduce the cost of the beam.

The paper first presents the methodology followed for the optimization of a beam designed as per ACI 318-19 using EA and its spreadsheet implementation. The results of three different design examples are presented next to prove the effectiveness of the implementation. Lastly, the results of a detailed parametric study conducted using the developed spreadsheet tool are presented.

## 2. Methodology for Spreadsheet Optimization

In this study, a spreadsheet-based tool is developed for the optimal design of RC beams. The spreadsheet-based optimization is comparatively easier to use for civil engineers as compared to script-based optimization using a programming language. The spreadsheet in this study follows the design procedure as per ACI 318-19. Initially, the objective function for the optimal design of a simply supported rectangular beam was formulated as discussed in the subsequent section. Afterwards, the design variables were set for this study. This also included the strength and serviceability constraints for those variables as per ACI-318 provisions. Once the formulation of the problem was done, an algorithm for economical design was prepared in MS Excel. All the possible design trials/iterations are run in the Excel Sheet using a built-in add-in, the “Solver” tool. The Evolutionary Algorithm (EA), which is a better-suited technique for beam design optimization-type non-smooth and non-convex problems [16], is used to achieve the most economical solution while fulfilling all the design requirements.

### 2.1. Objective Function

The cost-based function provides the best results for concrete [19]. Four factors$—C_{c}$ (cost of concrete), $V_{c}$ (volume of concrete), $C_{s}$ (cost of steel) and $W_{st}$ (weight of steel)—have been considered in the cost objective function. The material costs (in PKR) are taken from local contractors as PKR 9167 per cubic meter (cu.m) for 20 MPa concrete, and PKR 120 and 135 per kg for reinforcing bars of 275 MPa and 414 MPa, respectively. The cost function $f\left(cost\right)$ is defined in Equation (1).

(1) $$f\;\left(cost\right)=C_{s}\cdot W_{st}+C_{c}\cdot V_{c}$$

It is imperative to note that the volume of concrete ($V_{c}$) has been calculated by the product of the width, depth, and span of the beam. While computing this volume, an adjustment for the steel reinforcement was made by subtracting the reinforcement volume, i.e., $A_{s}\times L$, where $A_{s}$ represents the area of steel in the beam and *L* denotes the beam span. A concrete cover of 40 mm was used in the volume computations.

### 2.2. Design Variables 

In total, there are three design variables, namely, the beam depth, width, and steel reinforcement. All these variables are provided with their upper and lower bounds to avoid infeasible sections. The bounds are collected through literature reviews and through the ACI 318 provisions for the minimum allowable dimensions. For instance, the lower bound for width (b), i.e., 228 mm, is based on local practices adopted in Pakistan, and the lower bound for beam depth (d) is in accordance with the ACI-318 provisions. However, the upper bounds for these variables are user-defined. The maximum and minimum steel reinforcement ratios ($\rho_{max}$ and $\rho_{min}$) are as per ACI-318.

### 2.3. Constraints

Constraints are the specified conditions for parameters involved that must be obeyed to obtain values pertaining to structural requirements. They are defined by codes and ensure that the structure is within strength and serviceability limit states. For the optimal design of RC beams, the following constraints for design variables have been considered:

- The minimum permissible depth;
- The deflection control;
- The maximum and minimum area of steel;
- The failure type (under-reinforced section);
- The architectural constraints;
- The flexural capacity of the designed beam.

In order to optimize the design of an RC beam, several trials are to be run once the algorithm is ready. The design of the beam is selected based on the trial that provides the smallest value for the objective function. All the design variables are kept within some permissible upper and lower limiting values. Some such constraints are defined by the code provisions, e.g., the minimum and maximum area of steel, while some are defined by the user, e.g., the upper constraint for the depth of the beam, and the strengths of concrete and steel. The above-mentioned design constraints are further elaborated on in the sections below.

#### 2.3.1. Minimum Permissible Depth

The effective depth (d) of a beam is an important parameter in order to keep the deflections within the required limits. The minimum permissible depth of a beam depends on the required flexural strength of the beam and the allowable deflections in the beam. A generalized relation for the flexural requirement of a beam is expressed in Equation (2) [12].

(2) $$M_{u}=\varphi_{b}\times 0.85f_{c}^{\prime }\times b\times \frac{3}{8}\beta_{1}d_{min}\times \left(d_{min}-\frac{\frac{3}{8}\beta_{1}d_{min}}{2}\right)$$

where $\varphi_{b}$ is the Strength Reduction Factor for tension-controlled sections and $\beta_{1}$ represents the Whitney’s Stress Block Parameter. Generally, $\varphi_{b}$ is taken as 0.9, while the value of $\beta_{1}$ is affected by 28 days compressive strength of concrete as:
$\beta_{1}=0.85$$\;For\;f_{c}^{\prime }\leq 28\;\mathrm{MPa}$$\beta_{1}=0.80$$\;For\;f_{c}^{\prime }=35\;\mathrm{MPa}$$\beta_{1}=0.75$$\;For\;f_{c}^{\prime }=41\;\mathrm{MPa}$$\beta_{1}=0.70$$\;For\;f_{c}^{\prime }=48\;\mathrm{MPa}$$\beta_{1}=0.65$$\;For\;f_{c}^{\prime }\geq 55\;\mathrm{MPa}$

For instance, as a part of this study, the minimum effective depth of a singly reinforced simply supported beam ($d_{min}$) for $f_{c}^{\prime }\leq 28\;\mathrm{MPa}$ is expressed in Equation (3).

(3) $$d_{min}=\sqrt{\frac{M_{u}}{0.205\times f_{c}^{\prime }\times b}}\;\;\;\;\;\;\;\;\;\;\;\;For\;f_{c}^{\prime }\leq 28\;\mathrm{MPa}$$

Here, $M_{u}$ is the required factored moment, b is the width of the beams, and $f_{c}^{\prime }$ is the 28-days compressive strength of concrete. It must be kept in mind that a concrete cover of 40 mm (as suggested by ACI 318) has been considered in the design exercise. The total volume of the concrete material is calculated for the overall depth (d+40) of the beam.

#### 2.3.2. ACI Minimum Reinforcement

Lesser steel may lead to a brittle failure of the member without providing sufficient warning before the failure. This type of failure is undesirable. To ensure against this type of failure, a lower limit must be put in place by equating the cracking moment. ACI-318 recommends the minimum value of the steel reinforcement ratio $\left(\rho_{min}\right)$ for the beam as given in Equation (4).

(4) $$\rho_{min}=0.25\times \frac{\sqrt{f_{c}^{\prime }}}{f_{y}}\geq \frac{1.4}{f_{y}}$$

In Equation (4), $f_{y}$ is the yield strength of reinforcing steel and $f_{c}^{\prime }$ is the compressive strength of concrete.

Following the basic steel ratio definition, i.e., $\rho =A_{s}/bd$, the minimum area of steel ($A_{s,min}$) is as given in Equation (5). The second expression in Equation (5) governs when $f_{c}^{\prime }\leq 31.4\;\mathrm{MPa}$

(5) $$A_{s,\;min}=0.25\times \frac{\sqrt{f_{c}^{\prime }}}{f_{y}}\times b_{w}\times d\geq \frac{1.4}{f_{y}}b_{w}\times d$$

where $b_{w}$ is the width of the beam web and d is the depth of the beam.

For optimizing the quantity of reinforcement in the beam, the design constraint of the steel area ($A_{s}$) as given in Equation (6) was considered with respect to a minimum steel area ($A_{s,min}$).

(6) $$1-\frac{A_{s}}{A_{s,min}}\leq 0$$

#### 2.3.3. Maximum Steel Reinforcement ($\rho_{max}$)

To make sure the flexural member fails due to the yielding of the tension steel and provides sufficient warning before failure, ACI 9.3.1 requires that if the axial load on a non-prestressed member is less than $0.10f_{c}^{\prime }A_{g}$ ($A_{g}$ being the gross area of the structural element), then strain in steel is larger than 0.005, and it can be ensured when the maximum reinforcement ratio $\left(\rho_{max}\right)$ is as given in Equation (7)

(7) $$\rho_{max}=0.85\frac{f_{c}^{\prime }}{f_{y}}\beta_{1}\frac{3}{8}$$

where $\beta_{1}$ is a parameter that represents the percentage of distance to the neutral axis when stress-strain block for concrete is converted into a simple rectangular geometric shape (Whitney’s Stress Block) for the simplicity of analysis. Following the basic steel ratio definition, i.e., $\rho =A_{s}/bd$, the maximum area of steel ($A_{s,max}$) is obtained using Equation (8).

(8) $$A_{s,max\;}=\rho_{max}\times b\times d$$

For optimizing the quantity of the reinforcement in the beam, the design constraint of steel area ($A_{s}$) as given in Equation (9) was considered with respect to a maximum steel area ($A_{s,max}$).

(9) $$\frac{A_{s}}{A_{s,max}}-1\leq 0$$

#### 2.3.4. Architectural Constraints

Architectural constraints may or may not be ensured by looking at the scenario. Sometimes the architectural restrictions will not allow for the changing of one dimension, e.g., the width of the beam (b) is taken equal to the width of support, and sometimes the upper range of the beam depth (d) is already known. As an example, if the permissible width of the beam is at least 228 mm then the design constraint of Equation (10) is applied when the iterative process is run by the algorithm.

(10) $$1-\frac{b}{0.228}\leq 0$$

#### 2.3.5. Flexural Constraint

The flexural check as given in Equation (11) is applied to check the capacity of the optimally designed section. The capacity must be greater than or equal to the applied moment.

(11) $$\varphi M_{n}\;\geq \;M_{u}$$

In Equation (11), φ is the strength reduction factor, $M_{n}\;$ is the nominal capacity of the optimally designed section of the beam, and $M_{u}$ is the required flexural capacity of the beam.

No shear reinforcement constraint was considered in the optimization process because shear ties in the beam are mostly governed by the minimum requirements. However, when there is significant shear forces in the beam, then the shear design will not be carried out with minimum requirements. Additionally, the cost of shear stirrups depends on the cross-section of the beam, and the increase in the cross-sectional dimensions of the beam would affect the overall cost owing to an increase in the steel for stirrups. However, in this study, considering the dominant stresses to be the flexural stresses, no shear design constraints have been considered in the design optimization and could be considered for future study. A summary of the design constraints used in the spreadsheet is given in Table 1.

### 2.4. Optimization Techniques

An Evolutionary Algorithm (EA) has been used in this study. Solver, a built-in MS Excel optimization tool, is used in the spreadsheet. Solver is easily available as an add-in tool for the familiar MS Excel. To ease its application, pre-set settings have been utilized and Macros have been applied to create a simple interface. The settings used for the heuristic technique of EA in the spreadsheet include a population size of 100, a cross-over rate of 0.5, and a mutation rate of 0.1. Due to a “Random Seed” providing different results for each trial, a strategy was developed to apply the Generalized Reduced Gradient (GRG) before running the Evolutionary Algorithm (EA), to bring the initial solution closer to the global optimal.

### 2.5. Spreadsheet Implementation

**Figure 1:**
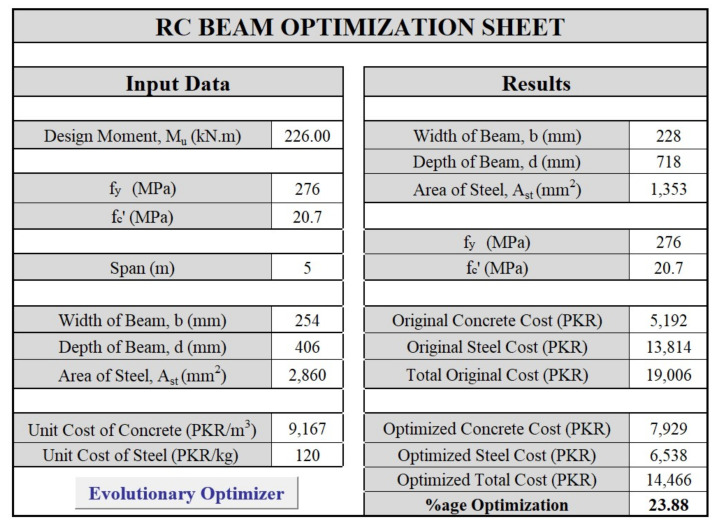
The interface for the RC beam optimizer.

The optimization is applied on reinforced concrete beams designed in prominent literature using the objective function, variables, and constraints presented in the above sections. The parameters utilized and the optimization sheet are shown in Figure 1, in which the simplicity of the developed sheet’s interface is evident.

## 3. Results and Discussion

### 3.1. Case Studies

Three singly reinforced beam examples collected from the prominent literature [20,21,22] have been optimized to demonstrate the effectiveness of the optimization approach adopted in this study.

#### 3.1.1. Example 1

Determine the cross-section of a beam and cross-section area of rebar required to resist a factored moment of 189 kN·m. The simply supported beam has a span of 4.57 m. Concrete having a compressive strength of 4000 psi (27.5 MPa) and rebars having a yield strength of 60,000 psi (414 MPa) are to be used [20].

Table 2 shows the results of the original design proposed in the book in comparison to the optimized design obtained using the Evolutionary Algorithm. The total cost of the optimized beam cross-section is approximately 20% less than the original design.

#### 3.1.2. Example 2

In a slab system, longer beams have a single simply supported effective span of 8 m and shorter beams have three spans of 5 m each. The slab thickness is 160 mm and the floor finishes consist of 75 mm of brick ballast and 50 mm of floor finish. The longer beams support a 228-mm-thick and a 3-m-high wall. The structure is to be used as an office building. Use C-20 concrete and grade 420 steel. Selecting the US bars, design the interior longer beam having a rectangular section width of 300 mm for flexure under the following conditions [21].

Table 3 presents the comparison of the results of the original design proposed in the book and the optimized design obtained using the EA. The total cost of the optimized beam cross-section is approximately 8% less than the original design.

#### 3.1.3. Example 3

Determine the cross-section of concrete and area of steel required for a simply supported beam with a span of 15 ft (4.572 m), which is to carry a computed dead load of 1.27 kip/ft (18.5 kN/m) and service live load of 2.44 kip/ft (35.6 kN/m). A 3000 psi (20.7 MPa) concrete is to be used, and the steel yield strength is 40,000 psi (276 MPa) [22].

A comparison of the results as proposed in the book and those obtained by optimization is presented in Table 4. A reduction of 24% in the total cost of the optimized beam is obtained as compared to the original design.

It is evident from the above examples that the developed tool gives an optimal solution with a single click. The tool runs all the feasible solutions for the beam design and picks the one that has the minimum construction cost. General trends from the solved examples show that in each optimized solution, the depth of the beam is taken higher than the author’s suggested value, whereas the area of steel is reduced. This implies that an extra increase in steel area not only gives an expensive design of the beam but also reduces the ductility of the beam and hence the warning before failure. Therefore, it is suggested that a higher permissible value of the beam depth should be preferred instead of increasing the area of steel. All these optimal feasible solutions could be easily worked out with the help of the developed optimization tool instead of performing cumbersome manual calculations for several design options.

### 3.2. Parametric Study

In the second phase of this study, a parametric evaluation has been done to investigate the effect of different design variables on the economical design of the RC beam. The simply supported singly reinforced beam case of Example 3 has been considered for this parametric study due to it being the most common and general beam type encountered in practice. Several trials were run by changing only one variable at a time and keeping all others constant. Resultantly, the effect of various design parameters has been analyzed in detail to figure out the most economical range of a design variable. The unit costs for the materials used in the parametric study have been taken from the local vendors, as well as from the Market Rates System (MRS) published by the Government of Punjab (Pakistan) twice a year. All the unit costs used are given in Table 5.

This parametric interpretation helped in figuring out the ranges of the design variables that considerably affect the optimal construction cost of the beam. Once these upper and lower limits have been determined, a designer would be able to perform a self-assessment of whether his design is going to be economical and to what extent. So, without undertaking laborious computations, a design engineer could estimate the variables that needed to be modified to come up with an optimal and feasible design solution. The effects of all such prominent parameters are discussed in the subsequent sections.

#### 3.2.1. Effect of Beam Depth

**Figure 2:**
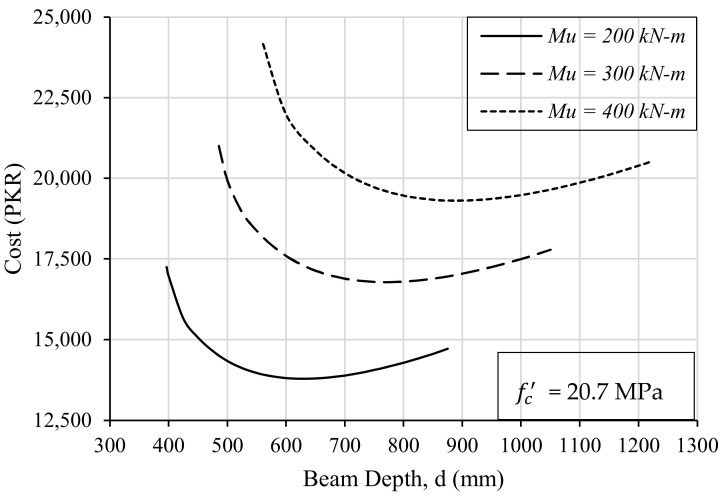
The relationship between the depth of the beam and the cost of construction.

To investigate the effect of the beam depth on the optimal design, around 400 trials were run for different values of depth of the beam against three flexural requirements ($M_{u}$). All the trials were run for the same values of concrete and steel strengths, i.e., $f_{c}^{\prime }$ = 20.7 MPa and $f_{y}$ = 276 MPa. The depth of the beam varied between the minimum required depth ($d_{\min}$) and the one where the ratio of the reinforcement in the beam reaches its minimum value ($\rho_{\min}$) for each loading case. The incurred cost against each increment of the beam depth was noted for every trial. The resulting data points were plotted by keeping the depth of the beam on the *x*-axis and the cost on the *y*-axis, as shown in Figure 2.

It is evident from Figure 2 that the value of loading does not directly affect the design optimization. The higher the value of the required flexural strength, the higher the cost of the construction of the beam will be. On the contrary, the depth of the beam affects the cost incurred in the construction of the beam. It is obvious from the graph that when the depth is kept equal to the minimum required value ($d_{\min}$), the cost of the beam is not minimal. Furthermore, the depth of the beam requires the maximum permissible ratio of steel ($\rho_{\max}$). Hence, this combination of $d_{\min}$ and $\rho_{\max}$ is not viable as it results in a higher cost of construction. A similar trend is also observed for maximum depth ($d_{\max}$) where the steel ratio is to be kept as the minimum allowed, i.e., $\rho_{\min}$.

**Figure 3:**
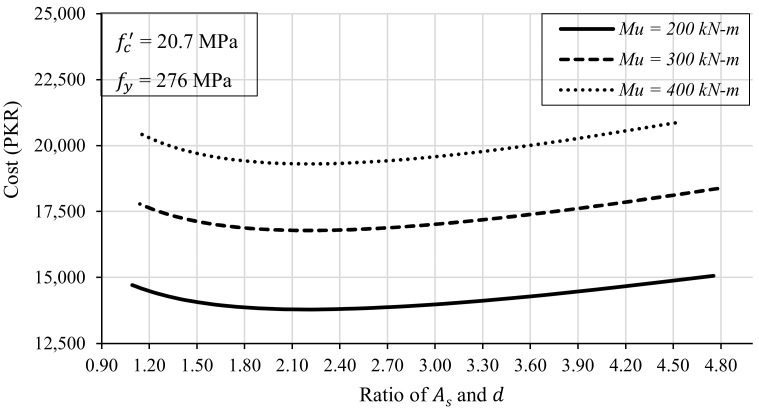
The effect of the ratio $A_{s}/d$ on the cost of the simply supported RC beam.

However, if the beam depth is kept somewhere between the minimum and maximum permissible depths ($d_{\min}$ and $d_{\max}$), the cost of construction is reduced. The most optimal solution lies almost in the middle of $d_{\min}$ and $d_{\max}$. It can be further observed that the width of the beam is kept as 228 mm, as chosen in the local practice, i.e., the equivalent to a two-brick-thick wall. Another significant trend was observed when the ratio of steel reinforcement ($A_{s}$) and depth of beam (d) was plotted against the beam cost. The most economical design of the beam results when the ratio $A_{s}/d$ lies in the range of 1.5 to 3 as shown in Figure 3. Therefore, structural engineers could cut the number of design trials by following this range during design optimization.

#### 3.2.2. Effect of Beam Width

**Figure 4:**
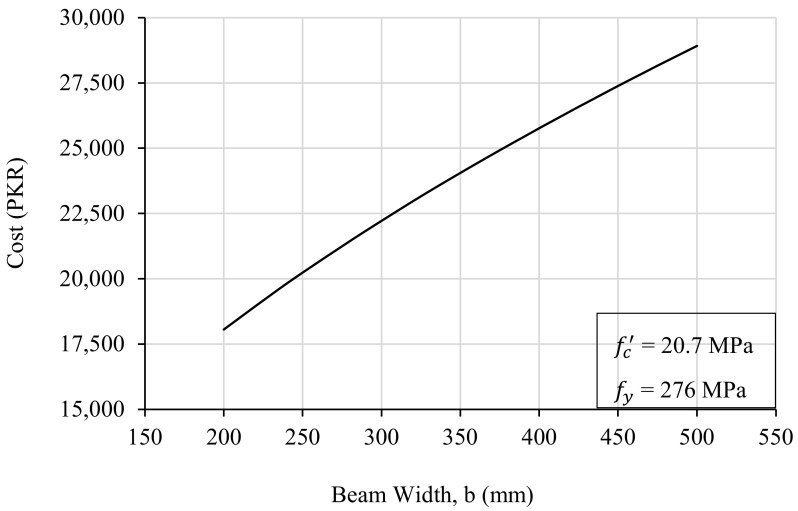
The effect of beam width on the cost of the beam.

In order to study the effect of beam width on the design optimization, 63 trials were run using the Excel sheet by changing the values of beam width (b) in each trial. Material strengths during these trials were kept as $f_{c}^{\prime }$ = 20.7 MPa and $f_{y}$ = 276 MPa. All the other design parameters were adjusted by the designed tool in each iteration, and the corresponding cost of the beam was noted. The width of the beam was varied from 200 mm to 500 mm, with an increase of 25 mm in each trial, and the resulting trend was plotted between the beam width on the abscissa and the incurred cost on the ordinate, as shown in Figure 4.

It can be observed from Figure 4 that there is a positive linear relationship between the width of the beam and its cost. The cost of the beam increases at a uniform rate with the increment in width of the beam. A nearly similar trend was obtained in all the trials. Therefore, it can be established that beam width does not have a notable effect on optimization. This is because it has a lesser impact on the design equations as compared to that of beam depth. Since beam width does not play a major role in the deflection control and reinforcement calculations, an increase in beam width will result in extra cost only. Due to these reasons, it is recommended to keep the beam width as minimal as possible. Hence, in common practice, the width of the beam is equal to the dimensions of the end supports.

#### 3.2.3. Effect of Reinforcement Ratio

**Figure 5:**
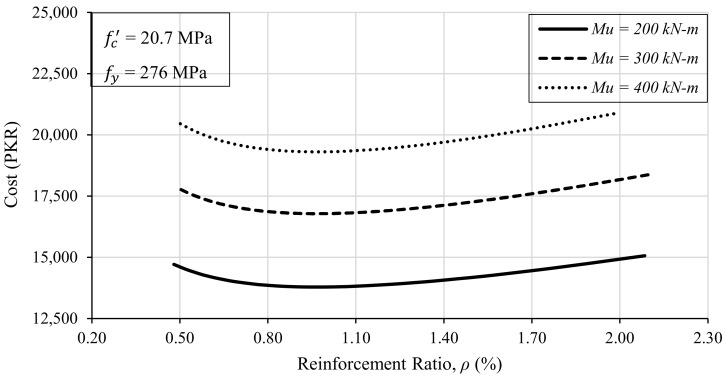
The effect of the reinforcement ratio (ρ) on the cost of the beam.

In order to study the effect of the reinforcement ratio on the economical design of the beam, 272 beam design iterations were run for different values of reinforcement ratio (ρ) or area of steel ($A_{s}$). It must be noted that the strength of the materials ($f_{c}^{\prime }$ = 20.7 MPa and $f_{y}$ = 276 MPa) was kept the same in each trial. Furthermore, three different moment loadings were considered to investigate the effect of the steel ratio on the design optimization, if affected by the strength requirements. The other design variables, e.g., the depth and width of the beam were adjusted so that the flexural capacity of the designed beam became equal to the required strength. The obtained results were plotted by keeping the reinforcement ratio on the *x*-axis and the cost of construction of the beam on the *y*-axis, as shown in Figure 5.

It is evident from Figure 5 that the beam’s cost is directly related to the strength requirements. The higher the value of the required strength, the higher the cost of the construction will be. On the other hand, the cost of the beam varies nonlinearly with reinforcement ratio (ρ), i.e., the area of steel ($A_{s}$) in the beam. The cost of steel is higher at both the extremes of the steel ratio, i.e., $\rho_{\min}$ and $\rho_{\max}$. However, there is a range of steel reinforcement ratios for which the cost incurred on the construction of the beam is reduced to a minimum. It can be noticed that when the value of ρ is kept between 0.80% and 1.20%, the cost of the beam is much less than the cost at the extreme values of the reinforcement ratio (ρ). For example, the cost of the beam against $\rho_{\min}$ for a moment capacity of 300 kN-m is around PKR 17,900, and the same beam can be constructed at PKR 16,700 when the steel ratio is kept at around 1%. This difference of around 8% becomes very significant when several such beams are to be constructed on a multistorey building with the same floor plans. 

#### 3.2.4. Effect of Concrete Strength

**Figure 6:**
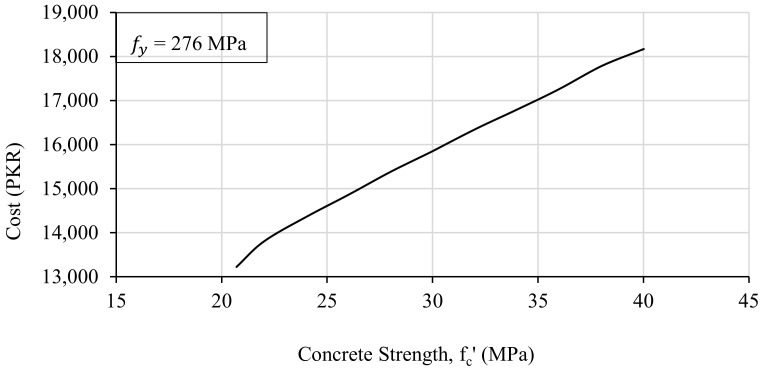
The effect of the concrete strength on the cost of the beam.

It is commonly established that the higher strength of concrete would reduce the cross-sectional dimensions of a reinforced concrete member and hence result in a lower cost of construction. To figure out this relationship between the cost of construction and the compressive strength of concrete, 22 trials were run to design the beam for different values of concrete strength ($f_{c}^{\prime }$) in each trial. The compressive strength of the concrete varied between 20.7 MPa and 40 MPa, and the corresponding unit cost was also adjusted for each strength value before each trial. All the other design parameters such as the width of the beam, the depth of the beam, and the required area of the steel were adjusted so that the resulting strength of the beam was greater than or equal to the required flexural strength. The obtained results are presented in the form of a graph by plotting the concrete strength on the abscissa and the cost of the construction on the ordinate, as shown in Figure 6.

Figure 6 shows that there is a linear relationship between the construction cost of a beam and the concrete compressive strength. Cost increases at a uniform rate with the increase in the compressive strength of concrete. Although the higher strength of the concrete results in a smaller cross-section of the beam and hence requires less concrete, the unit cost of high-strength concrete is much higher than the normal-strength concrete. Therefore, the difference in cost, which is obtained due to the reduction of cross-sectional dimensions of the structural member, is invalidated by the increase in the unit cost of high-strength concrete.

**Figure 7:**
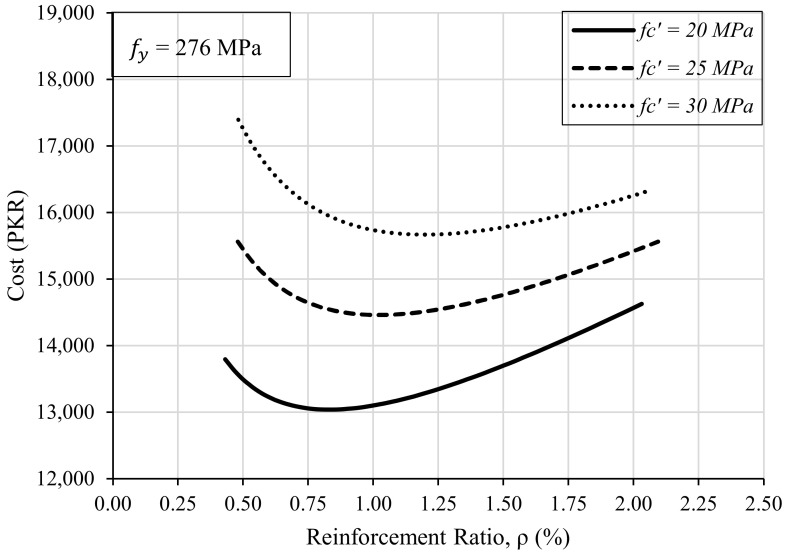
Effect of Compressive Strength of Concrete on Steel Ratio.

Furthermore, it was also studied whether the increase in concrete strength affects the required steel ratio or not. To do this, 144 trials were run by changing the steel ratio while keeping the loading and concrete strength, $f_{c}^{\prime }$ as the same. The depth and width of the beam are so adjusted in the spreadsheet that the flexural capacity of the designed beam is greater than or equal to the required capacity. The resulting data has been plotted in the form of a graph by taking the steel ratio on the *x*-axis and the cost of construction on the *y*-axis as shown in Figure 7. One curve for the variation of steel ratio was plotted for a single value of concrete strength.

Figure 7 shows that with the increase in compressive strength of concrete, the relationship between the steel ratio and the incurred cost of construction remains almost similar. However, high-strength concrete results in higher construction cost. It is because high-strength concrete requires controlled conditions for the preparation and transportation of concrete. Therefore, the use of normal-strength concrete (18–21 MPa) is recommended for ordinary construction unless otherwise required.

**Figure 8:**
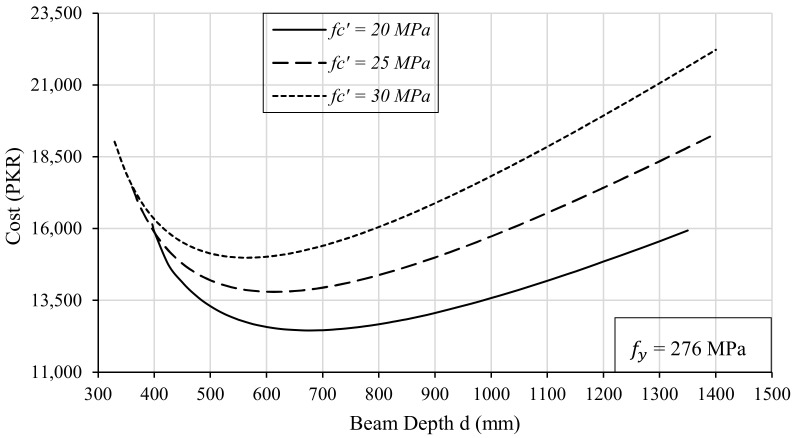
Effect of Compressive Strength of Concrete on Beam Depth (d).

The compressive strength of the concrete also affects the depth of an RC beam when it comes to an optimization problem. It can be seen from Figure 8 that for a higher depth of the beam, the difference in the construction cost varies at a higher rate for different grades of concrete. Furthermore, the range of the beam depth is not fixed for different grades of concrete. For higher-strength concretes, an optimized design can be achieved with lower depths than those of the lower grades of concrete. It must be noted that all these curves have been generated against a fixed value of bending moment ($M_{u}$), i.e., 226 kN-m. Ranges of the optimal solution for depth will be directly affected by an increase or decrease in loading on the beam but with a somewhat similar trend, as shown in Figure 8.

#### 3.2.5. Effect of Steel Strength

**Figure 9:**
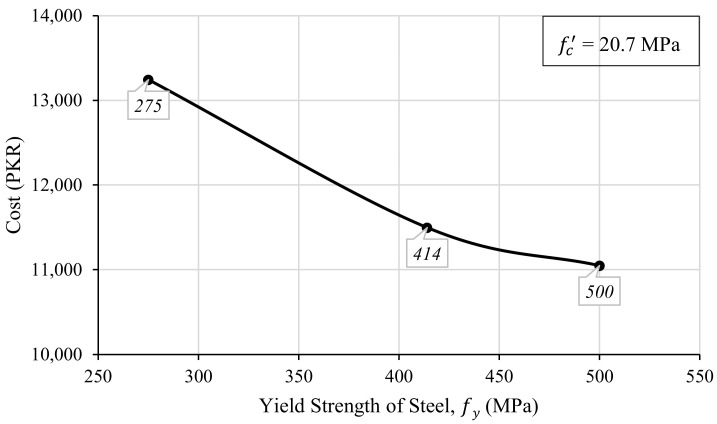
The relationship between the yield strength of the steel and the construction cost of the beam.

Steel is an important ingredient of reinforced concrete members. Plain concrete is weak in tension, so steel is used to strengthen concrete by providing strength against tensile forces that are produced due to flexural stresses. A major part of the cost of reinforced concrete is comprised of the cost of steel reinforcement. Therefore, it is very important to investigate the effect of steel strength on the design optimization of RC members. In order to do so, three commercially available steel strengths (275 MPa, 414 MPa, and 500 MPa) were selected, and 55 iterations were performed using the developed spreadsheet tool for each steel grade. During this iterative process, concrete strength was kept constant while all the other design parameters were adjusted so that the resulting strength of the beam was greater than or equal to the required flexural strength of the beam. The resulting data were plotted by keeping the steel strength on the abscissa and the construction cost of the beam on the ordinate, as shown in Figure 9. Although there is a minute difference in the density value of the three grades of steel investigated in this study, for the sake of simplicity, a fixed value of steel density 7850 kg/m^3^ is used.

It is obvious from Figure 9 that the optimization of the beam design increases with the increase in steel strength. This implies that when a higher grade of steel is used, the cost of construction is reduced. Since the ratio of steel used in the design mainly depends on the yield strength of steel, the higher steel strength requires a lesser quantity of steel to be used in the design. One could relate the reduced quantity to the higher unit cost of the high-grade steel, as in the case of concrete, but this would not be accurate because the unit cost of steel does not increase linearly with the increase in steel strength. With a small increment in the unit cost of steel, a higher strength margin is obtained. It is imperative to note that although higher strength provides the benefit of reduced steel demand in the design, the ability to provide a warning before the failure of the structure is reduced due to the lesser ductility of the high-strength steel.

**Figure 10:**
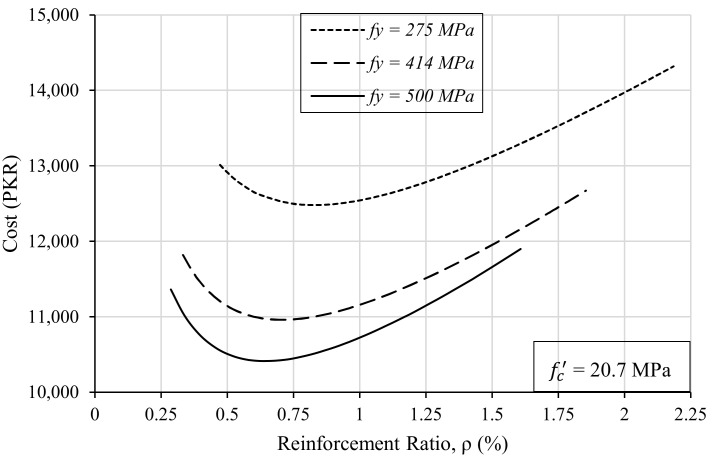
The effect of steel strength on the steel ratio and the cost of the beam.

This can be further elaborated with the help of another plot that is drawn between the amount of steel in the beam and the cost of construction of that beam. For this, 113 trials were performed using the same Excel sheet for different values of the steel ratio against a single grade of steel. This process was done for three different grades of steel having yield strengths of 275 MPa, 414 MPa, and 500 MPa. Then, the obtained results were plotted by keeping the steel ratio on the *x*-axis and the cost of construction of the beam on the *y*-axis, as shown in Figure 10.

Figure 10 shows that with the increase in steel strength, the required reinforcement ratio reduces and, consequently, the cost of the beam is also reduced. Therefore, it is recommended that the designers should also consider the use of higher grades of steel while designing reinforced concrete members. Although there lies a risk of reduced warning before failure due to the reduced ductility of higher-grade steels, it can be better investigated with further research.

**Figure 11:**
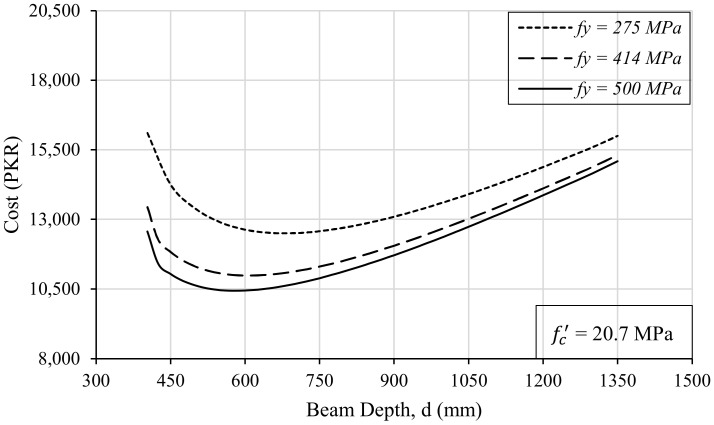
The effect of the steel strength on the depth and cost of the beam.

The strength of the steel has a direct relationship with the cost of the beam if plotted against the depth of the beam, as shown in Figure 11. For a single value of loading, e.g., 226 kN-m, 156 trials for beam design were run for three different grades of steel by changing the depth of the beam in each trial. While performing these trials, the compressive strength of the concrete and the width of the beam were kept constant. In this way, a direct relationship between beam depth and construction cost was obtained for different steel grades. It is obvious from Figure 11 that although the higher strength of steel results in the reduced cost of the beam, the range of optimal solutions for the depth of the beam remains almost similar. If all the other parameters are kept constant, a relatively optimal solution for beam depth would lie between 500 mm and 800 mm for this parametric study. In a similar way, if the constant values of the design parameters are changed, the corresponding optimal depth range can be easily figured out.

## 4. Conclusions

The optimization of the reinforced concrete members plays a pivotal role in the reduction of the overall cost. As a part of this study, a user-friendly beam design optimization tool has been developed using the Solver Add-on in the familiar MS Excel and using the EA. This tool has not only helped in performing a parametric study of various design parameters affecting RC beam design but has also resulted in an optimal solution after running several trials in the background. Major findings from this study carried out on optimization of RC beams are:

- There is a nonlinear relationship between the depth of the beam section and its cost of construction. The minimum required depth does not guarantee an economical design solution. The best design solution is obtained when the depth lies between two extreme ends, i.e., $d_{\min}$ and $d_{\max}$.
- The width of the beam section does not contribute to the optimization of the design. With the increase in beam width, the cost of construction increases linearly. Hence, an indefinite increase in the width of the beam will incur extra cost only.
- The ratio of steel reinforcement affects the optimization of the beam design. It has been concluded that when the ratio of steel lies within the range of 0.80% to 1.20%, the cost of beam construction is minimal as compared to that for the minimum and maximum values of the steel ratio.
- The probability of obtaining an economical solution is more when the ratio between the area of the steel and the depth of the beam is kept between 1.5 and 3.0.
- The use of high-strength concrete is not beneficial with respect to the cost optimization of the beam. Instead, normal-strength concrete is recommended to obtain an optimal solution.
- The grade of steel has an inverse relationship with the cost of the beam. A higher strength of steel requires less steel for the same flexural capacity and hence costs less. This is because the rate of increase in the steel cost with respect to its strength is much lower than the decrease in the cost of the beam against the high-strength steel.

It is pertinent to mention that the obtained optimal design solution would have continuous design data instead of the discrete values of the design variables as the tool is designed to provide the maximum level of optimization possible. It is required that the attained values of the design variables undergo an engineer’s examination before practical implementation, and the designer might need to round off the values to the nearest practical dimensions. To resolve this limitation, a stand-alone algorithm design is suggested for future studies using more design constraints related to practical aspects of the variables. Such an algorithm could be formulated using any programming tool (e.g., MATLAB) using a simple interface where the designer would be able to obtain practical design solutions with discrete data.

## Figures and Tables

**Table 1:** A summary of the constraints used in the spreadsheet.

Constraints	Governing Equation	Description
g_1_(x)	$1-\frac{A_{s}}{A_{s,min}}\leq 0$	Area of Steel
g_2_(x)	$\frac{A_{s}}{A_{s,max}}-1\leq 0$	
g_3_(x)	$1-\frac{b}{0.228}\leq 0$	Width of Beam
g_4_(x)	$1-\frac{d}{d_{min}}\leq 0$	Depth of Beam
g_5_(x)	$\varphi M_{n}\;\geq \;M_{u}$	Flexural Constraint

**Table 2:** The optimization results for Example 1.

Variables	Original	Optimized
Width of Beam, *b* (mm)	254	228
Depth of Beam, *d* (mm)	457	542
Area of Steel, $A_{st}$ (mm^2^)	1638	1004
Total Cost (PKR)	12,520	9987
% Optimization		20.20

**Table 3:** The optimization results for Example 2.

Variables	Original	Optimized
Width of Beam, *b* (mm)	300	300
Depth of Beam, *d* (mm)	702	876
Area of Steel, $A_{st}$ (mm^2^)	2716	2020
Total Cost (PKR)	23,321	21,355
% Optimization		8.43

**Table 4:** The optimization results for Example 3.

Variables	Original	Optimized
Width of Beam, *b* (mm)	254	228
Depth of Beam, *d* (mm)	406	718
Area of Steel, $A_{st}$ (mm^2^)	2860	1353
Total Cost (PKR)	17,379	13,223
% Optimization		24

**Table 5:** The unit costs of the materials used in the parametric study.

Material	Strength (MPa)	Unit	Cost (PKR)
Steel	275	per kg	120
	414		135
	500		145
Concrete	20	per cu.m	9167
	28		12,270
	30		13,059
	32		13,823
	34		14,650
	36		15,400
	38		16,190
	40		16,992

## References

[1] Mohammad F.A., Seyan D.A. (2016). Optimum design of reinforced concrete rectangular columns subjected to axial compression and biaxial bending moments. Athens J. Technol. Eng..

[2] Nusantara J.L.W., Aminullah A., Triwiyono A. (2019). Optimization of reinforced concrete column using android-based mobile application. MATEC Web of Conferences.

[3] Waheed J., Azam R., Riaz M.R., Shakeel M., Mohamed A., Ali E. (2022). Metaheuristic-Based Practical Tool for Optimal Design of Reinforced Concrete Isolated Footings: Development and Application for Parametric Investigation. Buildings.

[4] Kwak H.-G., Choi C., Chung G. (1996). Direct search approach to optimum spiral column design. Eng. Struct..

[5] Rabi’M N., Yousif S.T. (2014). Optimum Cost Design of Reinforced Concrete Columns Using Genetic Algorithms. AL Rafdain Eng. J..

[6] Bordignon R., Kripka M. (2012). Optimum design of reinforced concrete columns subjected to uniaxial flexural compression. Comput. Concr..

[7] Kanagasundaram S., Karihaloo B. (1991). Minimum-cost reinforced concrete beams and columns. Comput. Struct..

[8] Afzal M., Liu Y., Cheng J.C., Gan V.J. (2020). Reinforced concrete structural design optimization: A critical review. J. Clean. Prod..

[9] Rahmanian I., Lucet Y., Tesfamariam S. (2014). Optimal design of reinforced concrete beams: A review. Comput. Concr..

[10] Shakeel M., Azam R., Riaz M.R. (2022). A spreadsheet-based tool for optimal design of reinforced concrete cantilever retaining walls. Innov. Infrastruct. Solut..

[11] Nigdeli S.M., Bekdaş G., Yang X.-S. (2016). Application of the flower pollination algorithm in structural engineering. Metaheuristics and Optimization in Civil Engineering.

[12] ACI (2019). Building Code Requirements for Structural Concrete (ACI 318-19) and Commentary (ACI 318-19).

[13] Thomas S.M., Arulraj P. (2017). Optimization of singly reinforced RC beams. Int. J. Res.-Granthaalayah.

[14] Hisham Ajmal P. (2017). A study on cost optimized structural design of reinforced concrete beams. Int. J. Sci. Eng. Res..

[15] Raouache E., Logzit N., Driss Z., Khalfallah F. (2018). Optimization by RSM of reinforced concrete beam process parameters. Am. J. Mech. Eng..

[16] Correia R., Bono G., Bono G. (2019). Optimization of reinforced concrete beams using Solver tool. Rev. IBRACON Estrut. Mater..

[17] Ozimboski J.M., Pravia Z.M., Kripka M. (2020). Optimization of Reinforced Concrete Beams and Steel Beams: A Comparative Study. Int. J. Struct. Glass Adv. Mater. Res..

[18] Chutani S., Singh J. (2021). Use of constriction factor-based particle swarm optimization in design of reinforced concrete beams. Sustainable Development through Engineering Innovations: Select Proceedings of SDEI 2020.

[19] Coello C.C., Hernández F.S., Farrera F.A. (1997). Optimal design of reinforced concrete beams using genetic algorithms. Expert Syst. Appl..

[20] Darwin D., Dolan C.W., Nilson A.H. (2016). Design of Concrete Structures.

[21] Siddiqi Z.A. (2020). Concrete Structures, Part-I.

[22] Nilson A., Winter G. (1987). Design of Concrete Structures.

